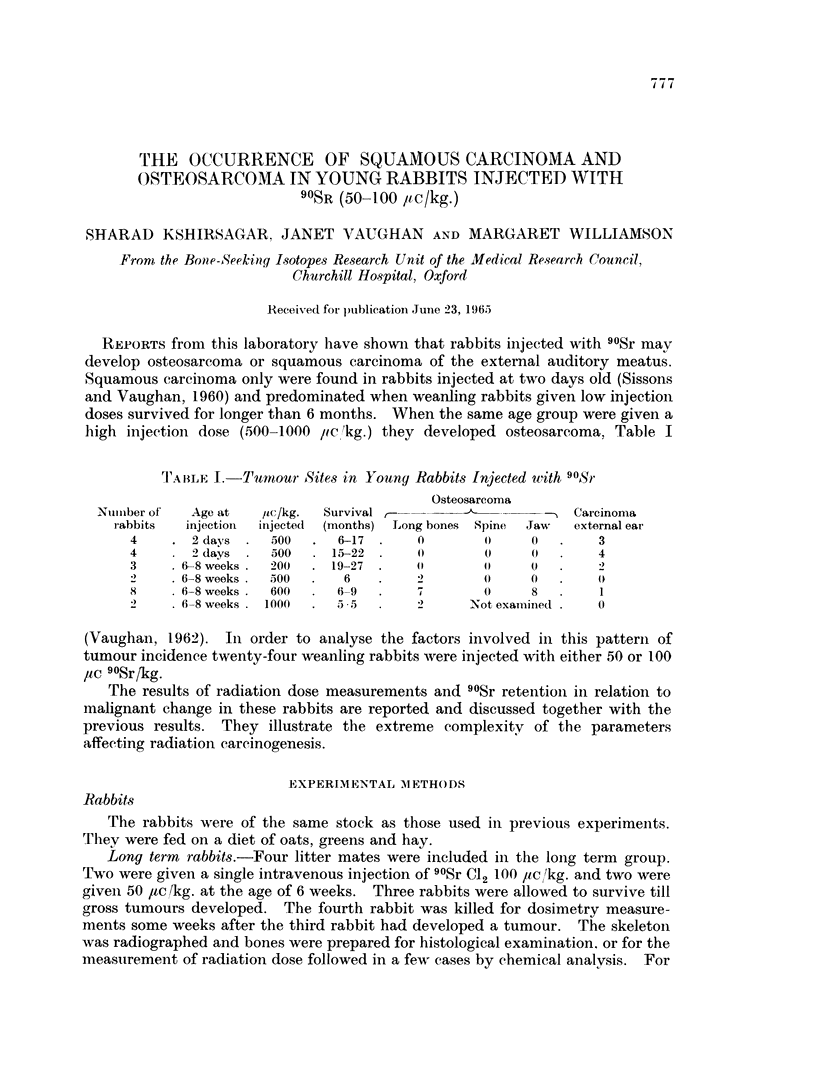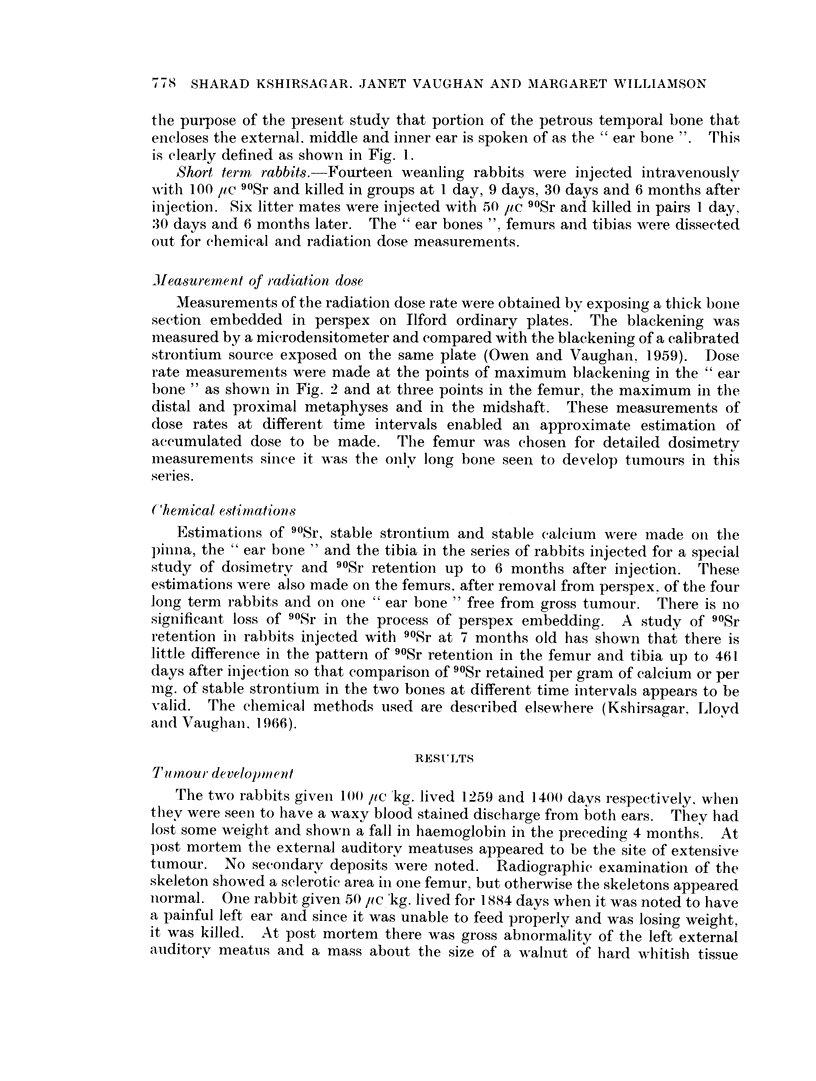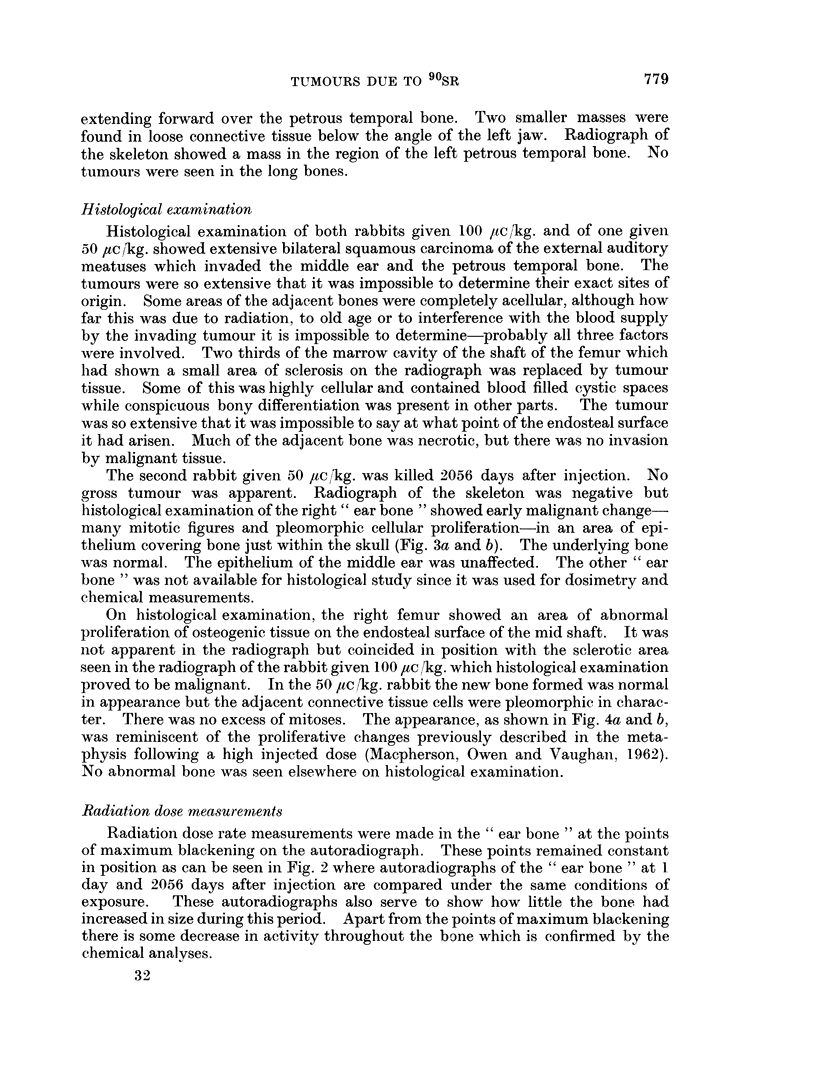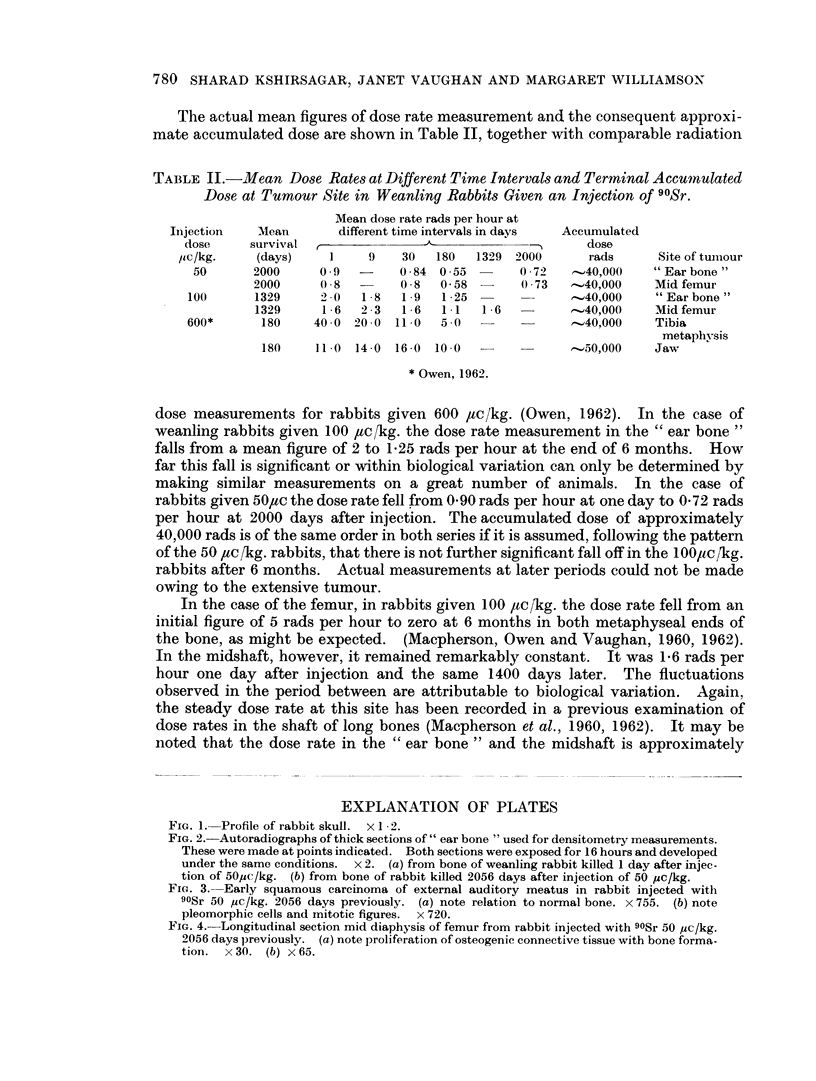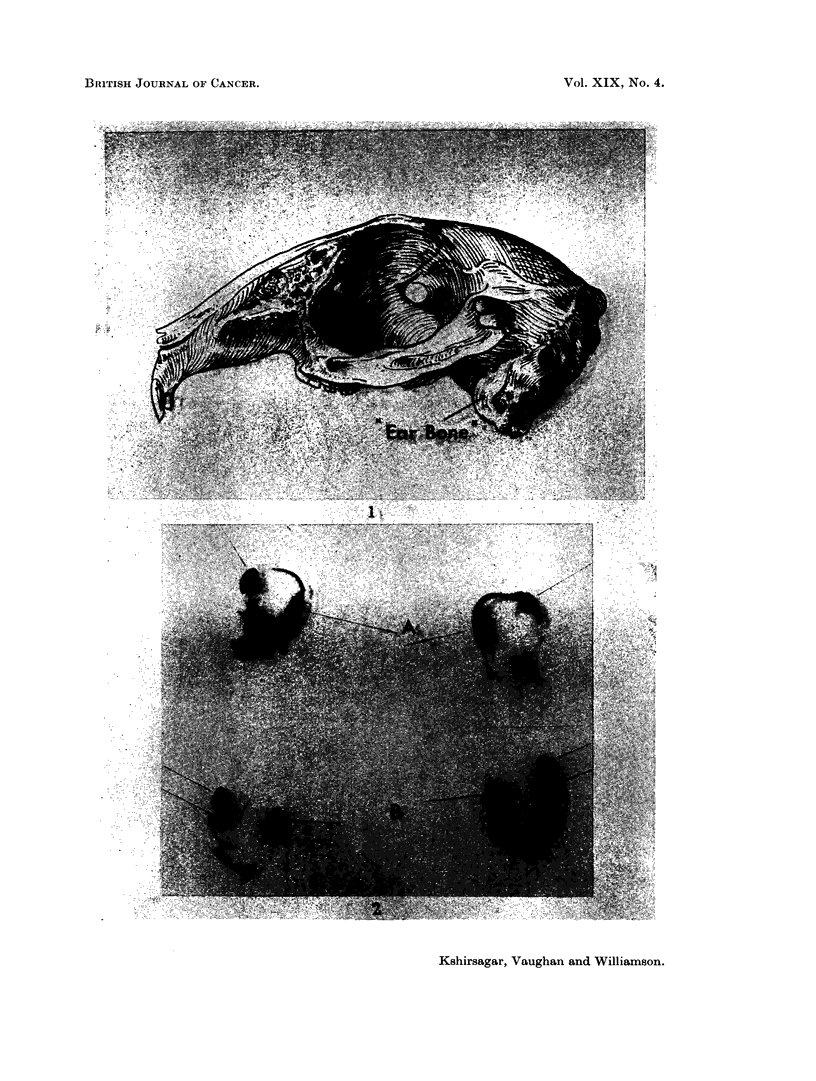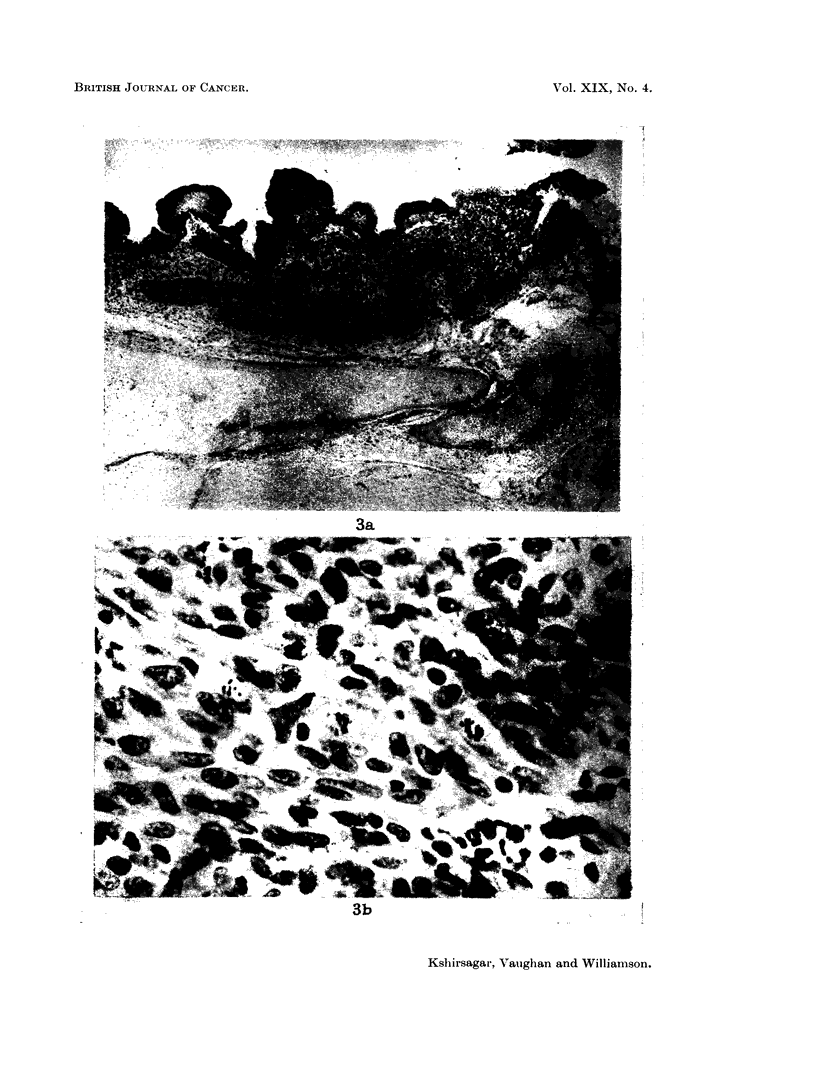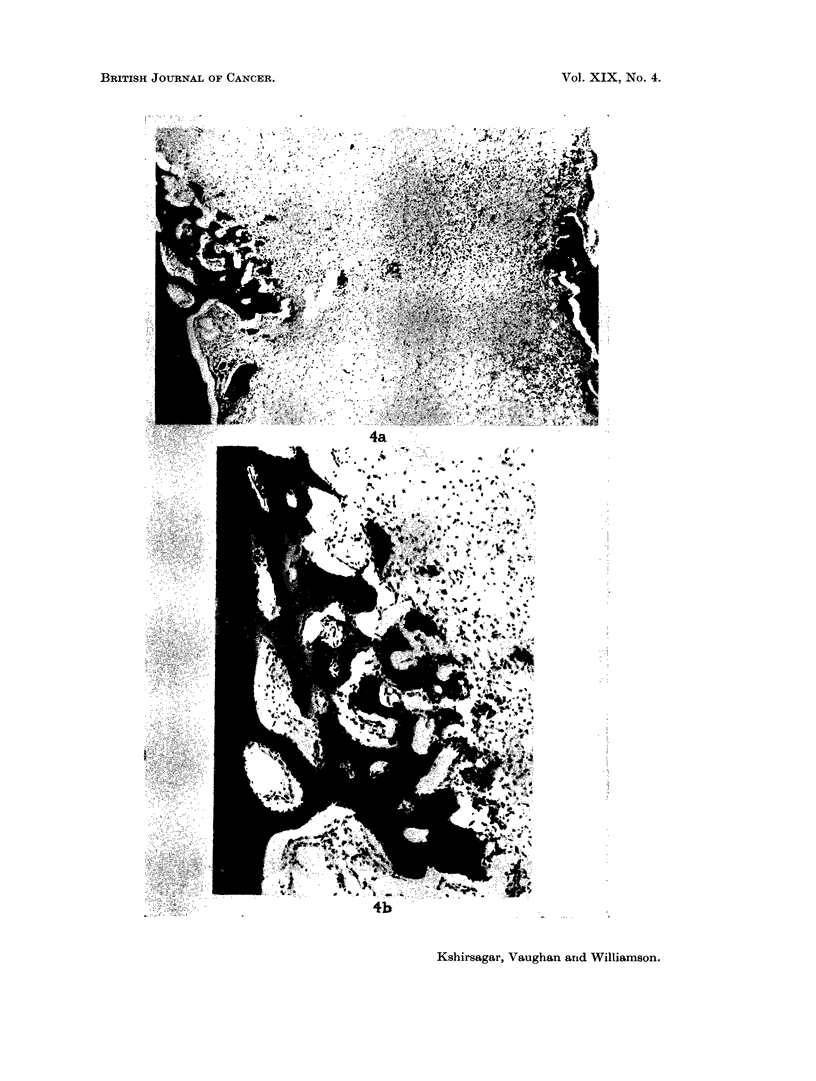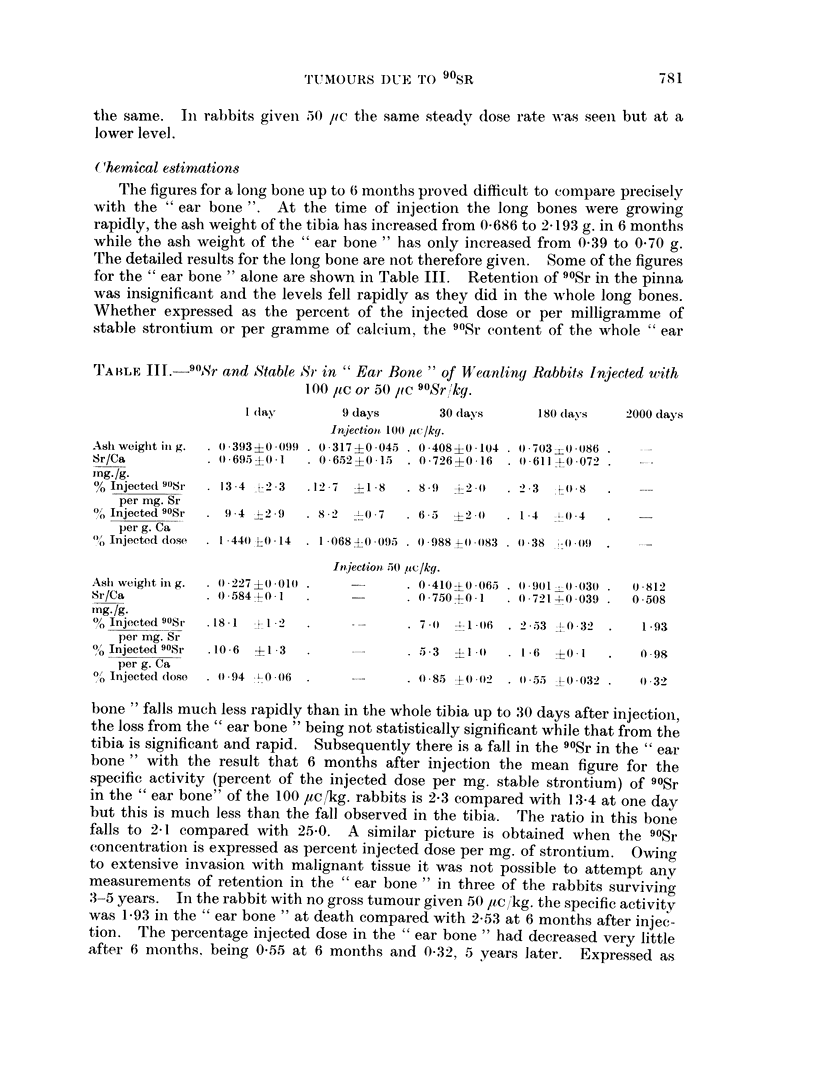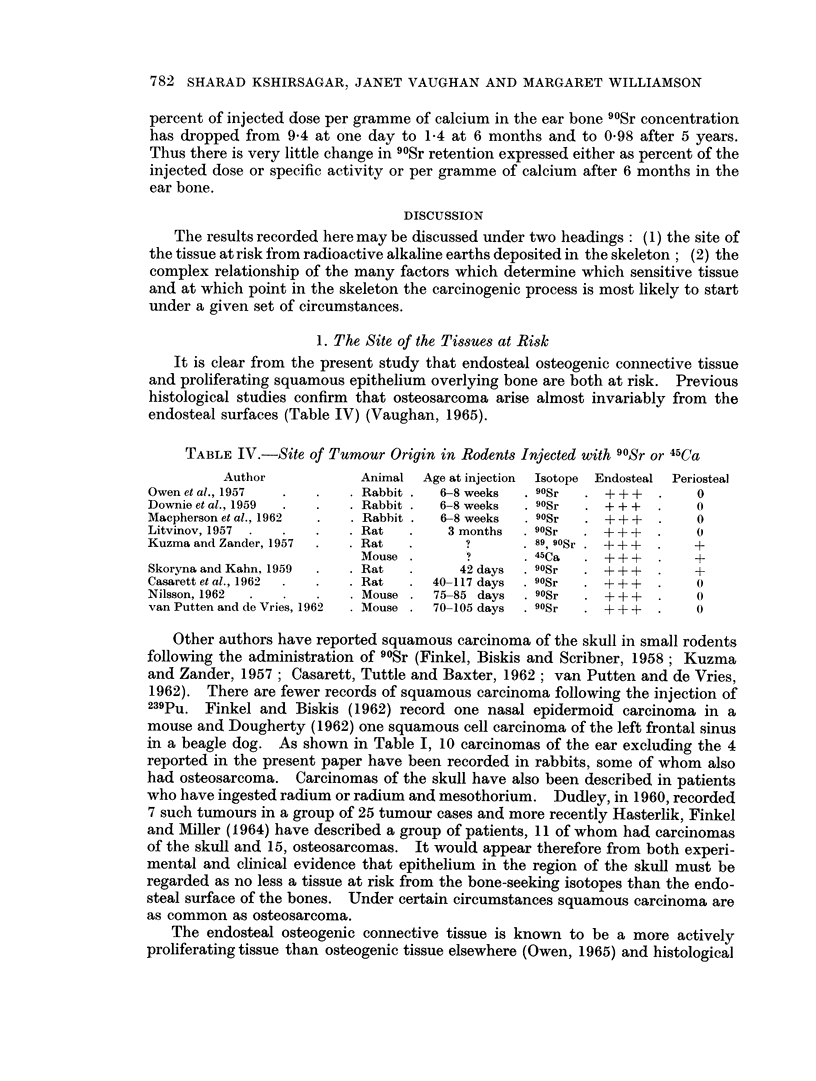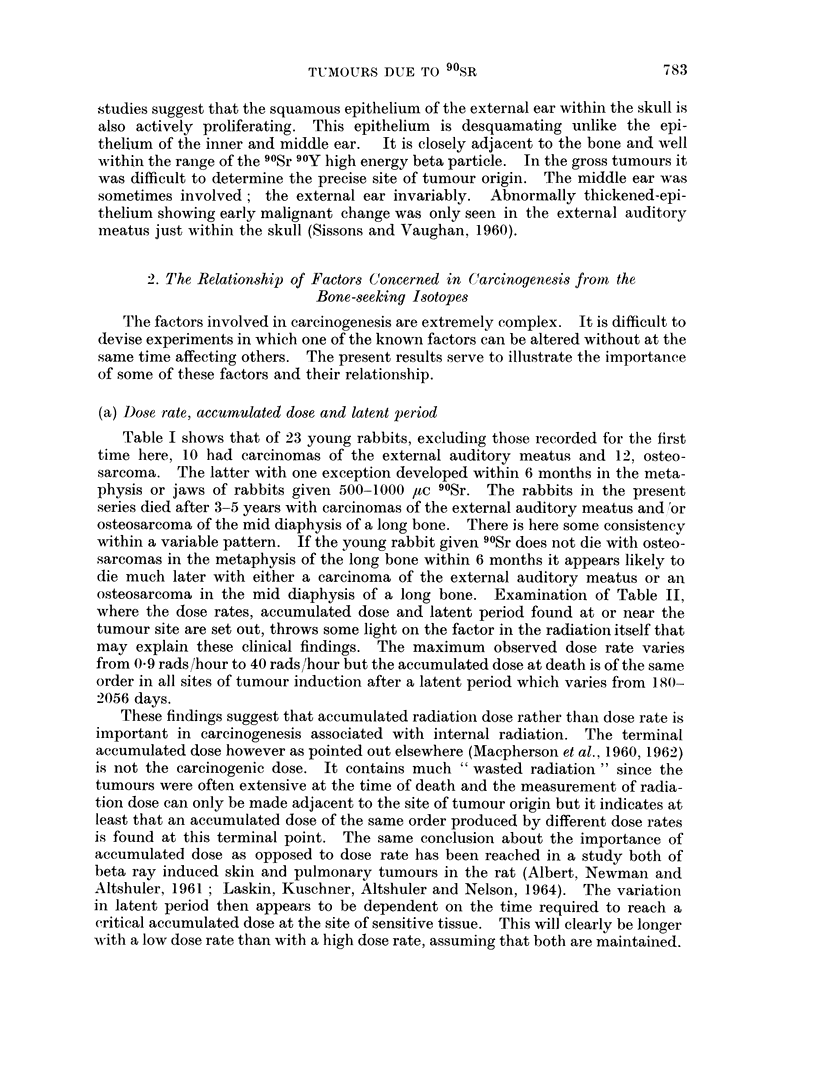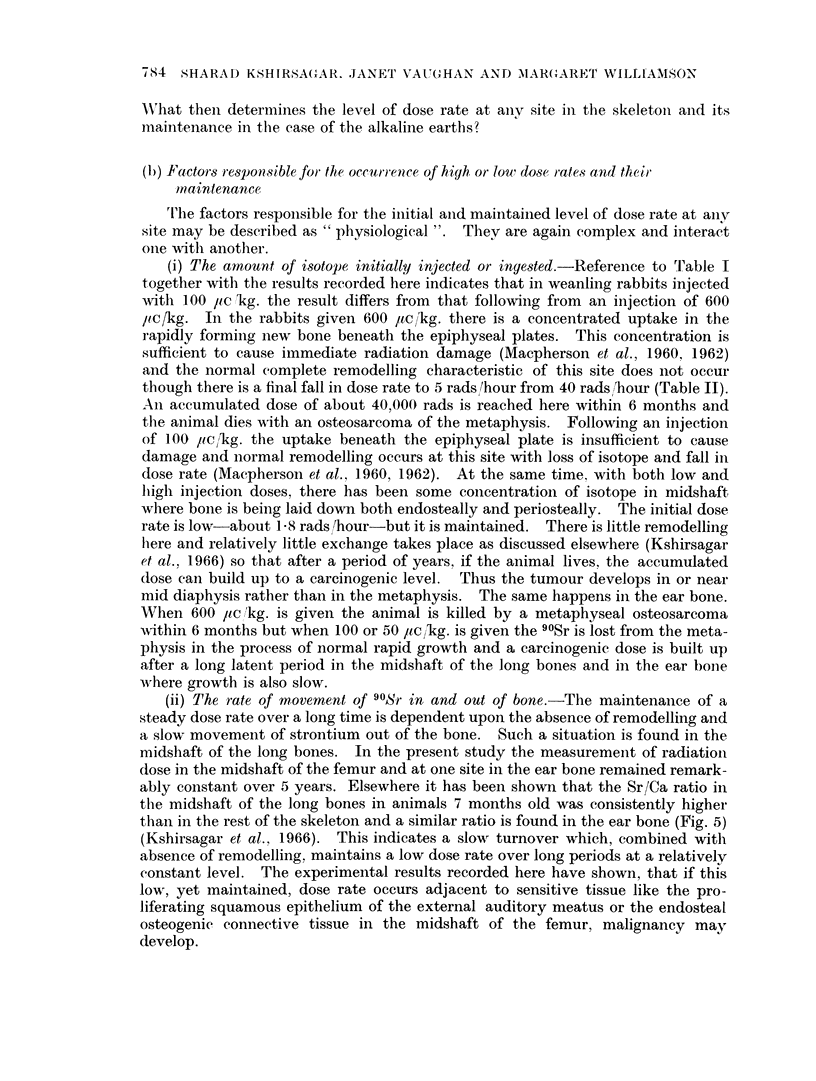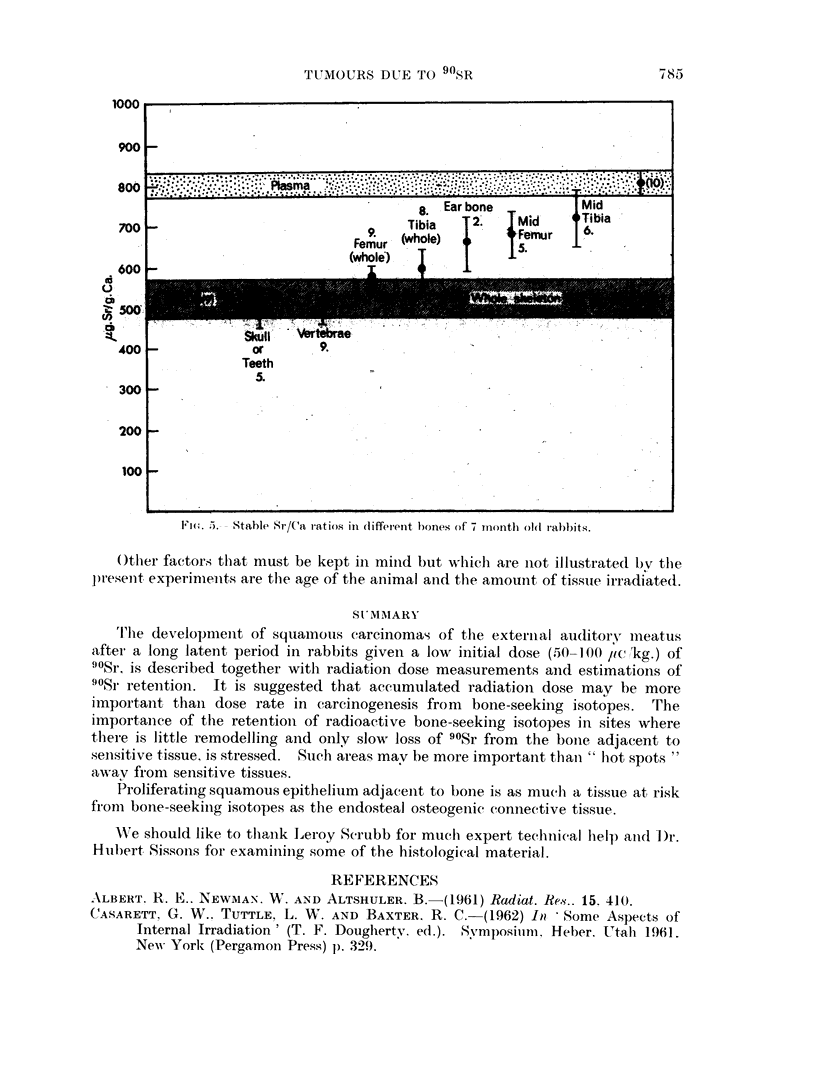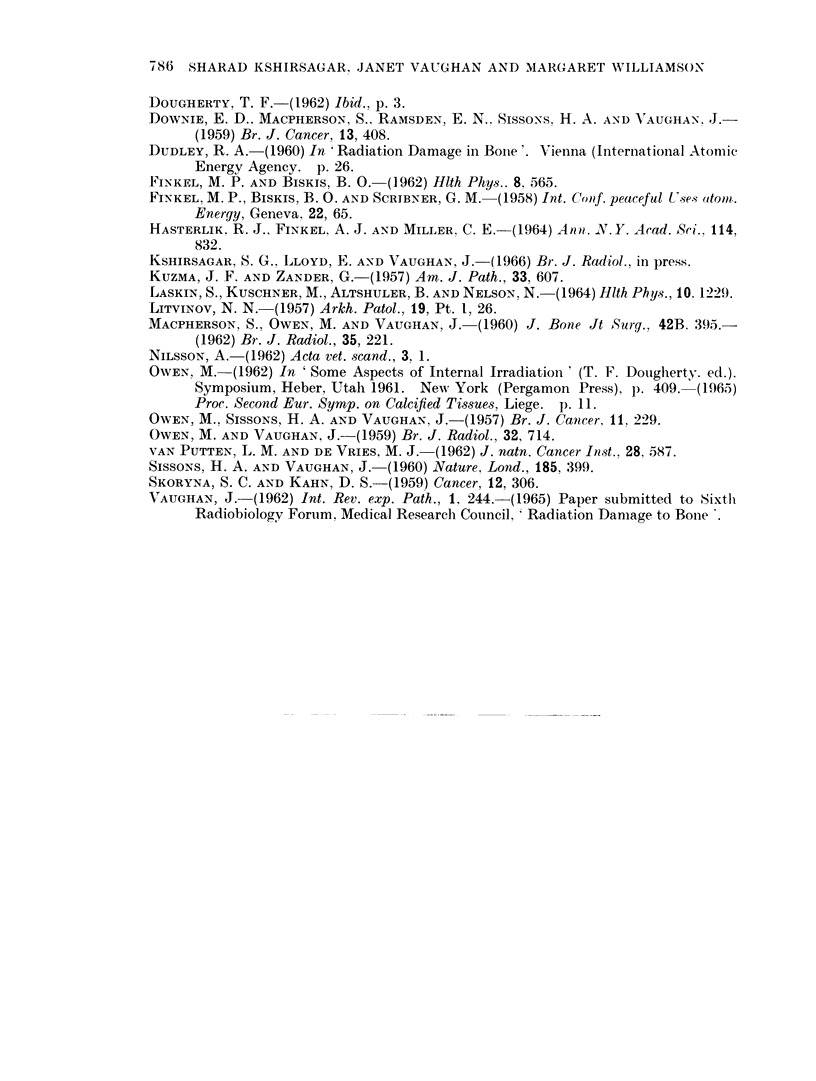# The occurrence of squamous carcinoma and osteosarcoma in young rabbits injected with 90Sr (50-100 mu-c--kg.).

**DOI:** 10.1038/bjc.1965.90

**Published:** 1965-12

**Authors:** S. Kshirsagar, J. Vaughan, M. Williamson

## Abstract

**Images:**


### THE OCCURRENCE OF SQUAMOUS CARCINOMA AND OSTEOSARCOMA IN YOUNG RABBITS INJECTED WITH $^{90}$Sr (50–100 $\mu$c/kg.)

SHARAD KSHIRSAGAR, JANET VAUGHAN AND MARGARET WILLIAMSON

*From the Bone-Seeking Isotopes Research Unit of the Medical Research Council, Churchill Hospital, Oxford*

Received for publication June 23, 1965

REPORTS from this laboratory have shown that rabbits injected with $^{90}$Sr may develop osteosarcoma or squamous carcinoma of the external auditory meatus. Squamous carcinoma only were found in rabbits injected at two days old (Sissons and Vaughan, 1960) and predominated when weanling rabbits given low injection doses survived for longer than 6 months. When the same age group were given a high injection dose (500–1000 $\mu$c/kg.) they developed osteosarcoma, Table I (Vaughan, 1962). In order to analyse the factors involved in this pattern of tumour incidence twenty-four weanling rabbits were injected with either 50 or 100 $\mu$c $^{90}$Sr/kg.

TABLE I.—*Tumour Sites in Young Rabbits Injected with $^{90}$Sr*

| Number of rabbits | Age at injection | $\mu$c/kg. injected | Survival (months) | Osteosarcoma: Long bones | Osteosarcoma: Spine | Osteosarcoma: Jaw | Carcinoma external ear |
|---|---|---|---|---|---|---|---|
| 4 | 2 days | 500 | 6–17 | 0 | 0 | 0 | 3 |
| 4 | 2 days | 500 | 15–22 | 0 | 0 | 0 | 4 |
| 3 | 6–8 weeks | 200 | 19–27 | 0 | 0 | 0 | 2 |
| 2 | 6–8 weeks | 500 | 6 | 2 | 0 | 0 | 0 |
| 8 | 6–8 weeks | 600 | 6–9 | 7 | 0 | 8 | 1 |
| 2 | 6–8 weeks | 1000 | 5·5 | 2 | Not examined | | 0 |

The results of radiation dose measurements and $^{90}$Sr retention in relation to malignant change in these rabbits are reported and discussed together with the previous results. They illustrate the extreme complexity of the parameters affecting radiation carcinogenesis.

### EXPERIMENTAL METHODS

### *Rabbits*

The rabbits were of the same stock as those used in previous experiments. They were fed on a diet of oats, greens and hay.

*Long term rabbits.*—Four litter mates were included in the long term group. Two were given a single intravenous injection of $^{90}$Sr $Cl_2$ 100 $\mu$c/kg. and two were given 50 $\mu$c/kg. at the age of 6 weeks. Three rabbits were allowed to survive till gross tumours developed. The fourth rabbit was killed for dosimetry measurements some weeks after the third rabbit had developed a tumour. The skeleton was radiographed and bones were prepared for histological examination, or for the measurement of radiation dose followed in a few cases by chemical analysis. For the purpose of the present study that portion of the petrous temporal bone that encloses the external, middle and inner ear is spoken of as the " ear bone ". This is clearly defined as shown in Fig. 1.

*Short term rabbits.*—Fourteen weanling rabbits were injected intravenously with 100 $\mu c$ $^{90}Sr$ and killed in groups at 1 day, 9 days, 30 days and 6 months after injection. Six litter mates were injected with 50 $\mu c$ $^{90}Sr$ and killed in pairs 1 day, 30 days and 6 months later. The " ear bones ", femurs and tibias were dissected out for chemical and radiation dose measurements.

*Measurement of radiation dose*

Measurements of the radiation dose rate were obtained by exposing a thick bone section embedded in perspex on Ilford ordinary plates. The blackening was measured by a microdensitometer and compared with the blackening of a calibrated strontium source exposed on the same plate (Owen and Vaughan, 1959). Dose rate measurements were made at the points of maximum blackening in the " ear bone " as shown in Fig. 2 and at three points in the femur, the maximum in the distal and proximal metaphyses and in the midshaft. These measurements of dose rates at different time intervals enabled an approximate estimation of accumulated dose to be made. The femur was chosen for detailed dosimetry measurements since it was the only long bone seen to develop tumours in this series.

*Chemical estimations*

Estimations of $^{90}Sr$, stable strontium and stable calcium were made on the pinna, the " ear bone " and the tibia in the series of rabbits injected for a special study of dosimetry and $^{90}Sr$ retention up to 6 months after injection. These estimations were also made on the femurs, after removal from perspex, of the four long term rabbits and on one " ear bone " free from gross tumour. There is no significant loss of $^{90}Sr$ in the process of perspex embedding. A study of $^{90}Sr$ retention in rabbits injected with $^{90}Sr$ at 7 months old has shown that there is little difference in the pattern of $^{90}Sr$ retention in the femur and tibia up to 461 days after injection so that comparison of $^{90}Sr$ retained per gram of calcium or per mg. of stable strontium in the two bones at different time intervals appears to be valid. The chemical methods used are described elsewhere (Kshirsagar, Lloyd and Vaughan, 1966).

### RESULTS

*Tumour development*

The two rabbits given 100 $\mu c$/kg. lived 1259 and 1400 days respectively, when they were seen to have a waxy blood stained discharge from both ears. They had lost some weight and shown a fall in haemoglobin in the preceding 4 months. At post mortem the external auditory meatuses appeared to be the site of extensive tumour. No secondary deposits were noted. Radiographic examination of the skeleton showed a sclerotic area in one femur, but otherwise the skeletons appeared normal. One rabbit given 50 $\mu c$/kg. lived for 1884 days when it was noted to have a painful left ear and since it was unable to feed properly and was losing weight, it was killed. At post mortem there was gross abnormality of the left external auditory meatus and a mass about the size of a walnut of hard whitish tissue extending forward over the petrous temporal bone. Two smaller masses were found in loose connective tissue below the angle of the left jaw. Radiograph of the skeleton showed a mass in the region of the left petrous temporal bone. No tumours were seen in the long bones.

*Histological examination*

Histological examination of both rabbits given 100 $\mu$c/kg. and of one given 50 $\mu$c/kg. showed extensive bilateral squamous carcinoma of the external auditory meatuses which invaded the middle ear and the petrous temporal bone. The tumours were so extensive that it was impossible to determine their exact sites of origin. Some areas of the adjacent bones were completely acellular, although how far this was due to radiation, to old age or to interference with the blood supply by the invading tumour it is impossible to determine—probably all three factors were involved. Two thirds of the marrow cavity of the shaft of the femur which had shown a small area of sclerosis on the radiograph was replaced by tumour tissue. Some of this was highly cellular and contained blood filled cystic spaces while conspicuous bony differentiation was present in other parts. The tumour was so extensive that it was impossible to say at what point of the endosteal surface it had arisen. Much of the adjacent bone was necrotic, but there was no invasion by malignant tissue.

The second rabbit given 50 $\mu$c/kg. was killed 2056 days after injection. No gross tumour was apparent. Radiograph of the skeleton was negative but histological examination of the right " ear bone " showed early malignant change—many mitotic figures and pleomorphic cellular proliferation—in an area of epithelium covering bone just within the skull (Fig. 3*a* and *b*). The underlying bone was normal. The epithelium of the middle ear was unaffected. The other " ear bone " was not available for histological study since it was used for dosimetry and chemical measurements.

On histological examination, the right femur showed an area of abnormal proliferation of osteogenic tissue on the endosteal surface of the mid shaft. It was not apparent in the radiograph but coincided in position with the sclerotic area seen in the radiograph of the rabbit given 100 $\mu$c/kg. which histological examination proved to be malignant. In the 50 $\mu$c/kg. rabbit the new bone formed was normal in appearance but the adjacent connective tissue cells were pleomorphic in character. There was no excess of mitoses. The appearance, as shown in Fig. 4*a* and *b*, was reminiscent of the proliferative changes previously described in the metaphysis following a high injected dose (Macpherson, Owen and Vaughan, 1962). No abnormal bone was seen elsewhere on histological examination.

*Radiation dose measurements*

Radiation dose rate measurements were made in the " ear bone " at the points of maximum blackening on the autoradiograph. These points remained constant in position as can be seen in Fig. 2 where autoradiographs of the " ear bone " at 1 day and 2056 days after injection are compared under the same conditions of exposure. These autoradiographs also serve to show how little the bone had increased in size during this period. Apart from the points of maximum blackening there is some decrease in activity throughout the bone which is confirmed by the chemical analyses.

The actual mean figures of dose rate measurement and the consequent approximate accumulated dose are shown in Table II, together with comparable radiation

TABLE II.—*Mean Dose Rates at Different Time Intervals and Terminal Accumulated Dose at Tumour Site in Weanling Rabbits Given an Injection of* $^{90}Sr$.

| Injection dose μc/kg. | Mean survival (days) | Mean dose rate rads per hour at different time intervals in days: 1 | 9 | 30 | 180 | 1329 | 2000 | Accumulated dose rads | Site of tumour |
|---|---|---|---|---|---|---|---|---|---|
| 50 | 2000 | 0·9 | — | 0·84 | 0·55 | — | 0·72 | ~40,000 | " Ear bone " |
| | 2000 | 0·8 | — | 0·8 | 0·58 | — | 0·73 | ~40,000 | Mid femur |
| 100 | 1329 | 2·0 | 1·8 | 1·9 | 1·25 | — | — | ~40,000 | " Ear bone " |
| | 1329 | 1·6 | 2·3 | 1·6 | 1·1 | 1·6 | — | ~40,000 | Mid femur |
| 600* | 180 | 40·0 | 20·0 | 11·0 | 5·0 | — | — | ~40,000 | Tibia metaphysis |
| | 180 | 11·0 | 14·0 | 16·0 | 10·0 | — | — | ~50,000 | Jaw |

* Owen, 1962.

dose measurements for rabbits given 600 μc/kg. (Owen, 1962). In the case of weanling rabbits given 100 μc/kg. the dose rate measurement in the " ear bone " falls from a mean figure of 2 to 1·25 rads per hour at the end of 6 months. How far this fall is significant or within biological variation can only be determined by making similar measurements on a great number of animals. In the case of rabbits given 50μc the dose rate fell from 0·90 rads per hour at one day to 0·72 rads per hour at 2000 days after injection. The accumulated dose of approximately 40,000 rads is of the same order in both series if it is assumed, following the pattern of the 50 μc/kg. rabbits, that there is not further significant fall off in the 100μc/kg. rabbits after 6 months. Actual measurements at later periods could not be made owing to the extensive tumour.

In the case of the femur, in rabbits given 100 μc/kg. the dose rate fell from an initial figure of 5 rads per hour to zero at 6 months in both metaphyseal ends of the bone, as might be expected. (Macpherson, Owen and Vaughan, 1960, 1962). In the midshaft, however, it remained remarkably constant. It was 1·6 rads per hour one day after injection and the same 1400 days later. The fluctuations observed in the period between are attributable to biological variation. Again, the steady dose rate at this site has been recorded in a previous examination of dose rates in the shaft of long bones (Macpherson *et al.*, 1960, 1962). It may be noted that the dose rate in the " ear bone " and the midshaft is approximately

### EXPLANATION OF PLATES

FIG. 1.—Profile of rabbit skull. ×1·2.

FIG. 2.—Autoradiographs of thick sections of " ear bone " used for densitometry measurements. These were made at points indicated. Both sections were exposed for 16 hours and developed under the same conditions. ×2. (*a*) from bone of weanling rabbit killed 1 day after injection of 50μc/kg. (*b*) from bone of rabbit killed 2056 days after injection of 50 μc/kg.

FIG. 3.—Early squamous carcinoma of external auditory meatus in rabbit injected with $^{90}Sr$ 50 μc/kg. 2056 days previously. (*a*) note relation to normal bone. ×755. (*b*) note pleomorphic cells and mitotic figures. ×720.

FIG. 4.—Longitudinal section mid diaphysis of femur from rabbit injected with $^{90}Sr$ 50 μc/kg. 2056 days previously. (*a*) note proliferation of osteogenic connective tissue with bone formation. ×30. (*b*) ×65.

1

2

Kshirsagar, Vaughan and Williamson.

3a

3b

Kshirsagar, Vaughan and Williamson.

4a

4b

Kshirsagar, Vaughan and Williamson.

the same. In rabbits given 50 $\mu$c the same steady dose rate was seen but at a lower level.

*Chemical estimations*

The figures for a long bone up to 6 months proved difficult to compare precisely with the " ear bone ". At the time of injection the long bones were growing rapidly, the ash weight of the tibia has increased from 0·686 to 2·193 g. in 6 months while the ash weight of the " ear bone " has only increased from 0·39 to 0·70 g. The detailed results for the long bone are not therefore given. Some of the figures for the " ear bone " alone are shown in Table III. Retention of $^{90}$Sr in the pinna was insignificant and the levels fell rapidly as they did in the whole long bones. Whether expressed as the percent of the injected dose or per milligramme of stable strontium or per gramme of calcium, the $^{90}$Sr content of the whole " ear

TABLE III.—*$^{90}$Sr and Stable Sr in " Ear Bone " of Weanling Rabbits Injected with 100 $\mu$c or 50 $\mu$c $^{90}$Sr/kg.*

| | 1 day | 9 days | 30 days | 180 days | 2000 days |
|---|---|---|---|---|---|
| | | *Injection 100 $\mu$c/kg.* | | | |
| Ash weight in g. | 0·393 ± 0·099 | 0·317 ± 0·045 | 0·408 ± 0·104 | 0·703 ± 0·086 | — |
| Sr/Ca mg./g. | 0·695 ± 0·1 | 0·652 ± 0·15 | 0·726 ± 0·16 | 0·611 ± 0·072 | — |
| % Injected $^{90}$Sr per mg. Sr | 13·4 ± 2·3 | 12·7 ± 1·8 | 8·9 ± 2·0 | 2·3 ± 0·8 | — |
| % Injected $^{90}$Sr per g. Ca | 9·4 ± 2·9 | 8·2 ± 0·7 | 6·5 ± 2·0 | 1·4 ± 0·4 | — |
| % Injected dose | 1·440 ± 0·14 | 1·068 ± 0·095 | 0·988 ± 0·083 | 0·38 ± 0·09 | — |
| | | *Injection 50 $\mu$c/kg.* | | | |
| Ash weight in g. | 0·227 ± 0·010 | — | 0·410 ± 0·065 | 0·901 ± 0·030 | 0·812 |
| Sr/Ca mg./g. | 0·584 ± 0·1 | — | 0·750 ± 0·1 | 0·721 ± 0·039 | 0·508 |
| % Injected $^{90}$Sr per mg. Sr | 18·1 ± 1·2 | — | 7·0 ± 1·06 | 2·53 ± 0·32 | 1·93 |
| % Injected $^{90}$Sr per g. Ca | 10·6 ± 1·3 | — | 5·3 ± 1·0 | 1·6 ± 0·1 | 0·98 |
| % Injected dose | 0·94 ± 0·06 | — | 0·85 ± 0·02 | 0·55 ± 0·032 | 0·32 |

bone " falls much less rapidly than in the whole tibia up to 30 days after injection, the loss from the " ear bone " being not statistically significant while that from the tibia is significant and rapid. Subsequently there is a fall in the $^{90}$Sr in the " ear bone " with the result that 6 months after injection the mean figure for the specific activity (percent of the injected dose per mg. stable strontium) of $^{90}$Sr in the " ear bone" of the 100 $\mu$c/kg. rabbits is 2·3 compared with 13·4 at one day but this is much less than the fall observed in the tibia. The ratio in this bone falls to 2·1 compared with 25·0. A similar picture is obtained when the $^{90}$Sr concentration is expressed as percent injected dose per mg. of strontium. Owing to extensive invasion with malignant tissue it was not possible to attempt any measurements of retention in the " ear bone " in three of the rabbits surviving 3–5 years. In the rabbit with no gross tumour given 50 $\mu$c/kg. the specific activity was 1·93 in the " ear bone " at death compared with 2·53 at 6 months after injection. The percentage injected dose in the " ear bone " had decreased very little after 6 months, being 0·55 at 6 months and 0·32, 5 years later. Expressed as percent of injected dose per gramme of calcium in the ear bone $^{90}Sr$ concentration has dropped from 9·4 at one day to 1·4 at 6 months and to 0·98 after 5 years. Thus there is very little change in $^{90}Sr$ retention expressed either as percent of the injected dose or specific activity or per gramme of calcium after 6 months in the ear bone.

### DISCUSSION

The results recorded here may be discussed under two headings : (1) the site of the tissue at risk from radioactive alkaline earths deposited in the skeleton ; (2) the complex relationship of the many factors which determine which sensitive tissue and at which point in the skeleton the carcinogenic process is most likely to start under a given set of circumstances.

### 1. *The Site of the Tissues at Risk*

It is clear from the present study that endosteal osteogenic connective tissue and proliferating squamous epithelium overlying bone are both at risk. Previous histological studies confirm that osteosarcoma arise almost invariably from the endosteal surfaces (Table IV) (Vaughan, 1965).

TABLE IV.—*Site of Tumour Origin in Rodents Injected with $^{90}Sr$ or $^{45}Ca$*

| Author | Animal | Age at injection | Isotope | Endosteal | Periosteal |
|---|---|---|---|---|---|
| Owen *et al.*, 1957 | Rabbit | 6–8 weeks | $^{90}Sr$ | +++ | 0 |
| Downie *et al.*, 1959 | Rabbit | 6–8 weeks | $^{90}Sr$ | +++ | 0 |
| Macpherson *et al.*, 1962 | Rabbit | 6–8 weeks | $^{90}Sr$ | +++ | 0 |
| Litvinov, 1957 | Rat | 3 months | $^{90}Sr$ | +++ | 0 |
| Kuzma and Zander, 1957 | Rat | ? | $^{89, 90}Sr$ | +++ | + |
| | Mouse | ? | $^{45}Ca$ | +++ | + |
| Skoryna and Kahn, 1959 | Rat | 42 days | $^{90}Sr$ | +++ | + |
| Casarett *et al.*, 1962 | Rat | 40–117 days | $^{90}Sr$ | +++ | 0 |
| Nilsson, 1962 | Mouse | 75–85 days | $^{90}Sr$ | +++ | 0 |
| van Putten and de Vries, 1962 | Mouse | 70–105 days | $^{90}Sr$ | +++ | 0 |

Other authors have reported squamous carcinoma of the skull in small rodents following the administration of $^{90}Sr$ (Finkel, Biskis and Scribner, 1958 ; Kuzma and Zander, 1957 ; Casarett, Tuttle and Baxter, 1962 ; van Putten and de Vries, 1962). There are fewer records of squamous carcinoma following the injection of $^{239}Pu$. Finkel and Biskis (1962) record one nasal epidermoid carcinoma in a mouse and Dougherty (1962) one squamous cell carcinoma of the left frontal sinus in a beagle dog. As shown in Table I, 10 carcinomas of the ear excluding the 4 reported in the present paper have been recorded in rabbits, some of whom also had osteosarcoma. Carcinomas of the skull have also been described in patients who have ingested radium or radium and mesothorium. Dudley, in 1960, recorded 7 such tumours in a group of 25 tumour cases and more recently Hasterlik, Finkel and Miller (1964) have described a group of patients, 11 of whom had carcinomas of the skull and 15, osteosarcomas. It would appear therefore from both experimental and clinical evidence that epithelium in the region of the skull must be regarded as no less a tissue at risk from the bone-seeking isotopes than the endosteal surface of the bones. Under certain circumstances squamous carcinoma are as common as osteosarcoma.

The endosteal osteogenic connective tissue is known to be a more actively proliferating tissue than osteogenic tissue elsewhere (Owen, 1965) and histological studies suggest that the squamous epithelium of the external ear within the skull is also actively proliferating. This epithelium is desquamating unlike the epithelium of the inner and middle ear. It is closely adjacent to the bone and well within the range of the $^{90}$Sr $^{90}$Y high energy beta particle. In the gross tumours it was difficult to determine the precise site of tumour origin. The middle ear was sometimes involved ; the external ear invariably. Abnormally thickened-epithelium showing early malignant change was only seen in the external auditory meatus just within the skull (Sissons and Vaughan, 1960).

### 2. *The Relationship of Factors Concerned in Carcinogenesis from the Bone-seeking Isotopes*

The factors involved in carcinogenesis are extremely complex. It is difficult to devise experiments in which one of the known factors can be altered without at the same time affecting others. The present results serve to illustrate the importance of some of these factors and their relationship.

(a) *Dose rate, accumulated dose and latent period*

Table I shows that of 23 young rabbits, excluding those recorded for the first time here, 10 had carcinomas of the external auditory meatus and 12, osteosarcoma. The latter with one exception developed within 6 months in the metaphysis or jaws of rabbits given 500–1000 $\mu$c $^{90}$Sr. The rabbits in the present series died after 3–5 years with carcinomas of the external auditory meatus and/or osteosarcoma of the mid diaphysis of a long bone. There is here some consistency within a variable pattern. If the young rabbit given $^{90}$Sr does not die with osteosarcomas in the metaphysis of the long bone within 6 months it appears likely to die much later with either a carcinoma of the external auditory meatus or an osteosarcoma in the mid diaphysis of a long bone. Examination of Table II, where the dose rates, accumulated dose and latent period found at or near the tumour site are set out, throws some light on the factor in the radiation itself that may explain these clinical findings. The maximum observed dose rate varies from 0·9 rads/hour to 40 rads/hour but the accumulated dose at death is of the same order in all sites of tumour induction after a latent period which varies from 180–2056 days.

These findings suggest that accumulated radiation dose rather than dose rate is important in carcinogenesis associated with internal radiation. The terminal accumulated dose however as pointed out elsewhere (Macpherson *et al.*, 1960, 1962) is not the carcinogenic dose. It contains much " wasted radiation " since the tumours were often extensive at the time of death and the measurement of radiation dose can only be made adjacent to the site of tumour origin but it indicates at least that an accumulated dose of the same order produced by different dose rates is found at this terminal point. The same conclusion about the importance of accumulated dose as opposed to dose rate has been reached in a study both of beta ray induced skin and pulmonary tumours in the rat (Albert, Newman and Altshuler, 1961 ; Laskin, Kuschner, Altshuler and Nelson, 1964). The variation in latent period then appears to be dependent on the time required to reach a critical accumulated dose at the site of sensitive tissue. This will clearly be longer with a low dose rate than with a high dose rate, assuming that both are maintained.

What then determines the level of dose rate at any site in the skeleton and its maintenance in the case of the alkaline earths?

(b) *Factors responsible for the occurrence of high or low dose rates and their maintenance*

The factors responsible for the initial and maintained level of dose rate at any site may be described as " physiological ". They are again complex and interact one with another.

(i) *The amount of isotope initially injected or ingested.*—Reference to Table I together with the results recorded here indicates that in weanling rabbits injected with 100 $\mu$c/kg. the result differs from that following from an injection of 600 $\mu$c/kg. In the rabbits given 600 $\mu$c/kg. there is a concentrated uptake in the rapidly forming new bone beneath the epiphyseal plates. This concentration is sufficient to cause immediate radiation damage (Macpherson *et al.*, 1960, 1962) and the normal complete remodelling characteristic of this site does not occur though there is a final fall in dose rate to 5 rads/hour from 40 rads/hour (Table II). An accumulated dose of about 40,000 rads is reached here within 6 months and the animal dies with an osteosarcoma of the metaphysis. Following an injection of 100 $\mu$c/kg. the uptake beneath the epiphyseal plate is insufficient to cause damage and normal remodelling occurs at this site with loss of isotope and fall in dose rate (Macpherson *et al.*, 1960, 1962). At the same time, with both low and high injection doses, there has been some concentration of isotope in midshaft where bone is being laid down both endosteally and periosteally. The initial dose rate is low—about 1·8 rads/hour—but it is maintained. There is little remodelling here and relatively little exchange takes place as discussed elsewhere (Kshirsagar *et al.*, 1966) so that after a period of years, if the animal lives, the accumulated dose can build up to a carcinogenic level. Thus the tumour develops in or near mid diaphysis rather than in the metaphysis. The same happens in the ear bone. When 600 $\mu$c/kg. is given the animal is killed by a metaphyseal osteosarcoma within 6 months but when 100 or 50 $\mu$c/kg. is given the $^{90}$Sr is lost from the metaphysis in the process of normal rapid growth and a carcinogenic dose is built up after a long latent period in the midshaft of the long bones and in the ear bone where growth is also slow.

(ii) *The rate of movement of $^{90}$Sr in and out of bone.*—The maintenance of a steady dose rate over a long time is dependent upon the absence of remodelling and a slow movement of strontium out of the bone. Such a situation is found in the midshaft of the long bones. In the present study the measurement of radiation dose in the midshaft of the femur and at one site in the ear bone remained remarkably constant over 5 years. Elsewhere it has been shown that the Sr/Ca ratio in the midshaft of the long bones in animals 7 months old was consistently higher than in the rest of the skeleton and a similar ratio is found in the ear bone (Fig. 5) (Kshirsagar *et al.*, 1966). This indicates a slow turnover which, combined with absence of remodelling, maintains a low dose rate over long periods at a relatively constant level. The experimental results recorded here have shown, that if this low, yet maintained, dose rate occurs adjacent to sensitive tissue like the proliferating squamous epithelium of the external auditory meatus or the endosteal osteogenic connective tissue in the midshaft of the femur, malignancy may develop.

FIG. 5.—Stable Sr/Ca ratios in different bones of 7 month old rabbits.

Other factors that must be kept in mind but which are not illustrated by the present experiments are the age of the animal and the amount of tissue irradiated.

### SUMMARY

The development of squamous carcinomas of the external auditory meatus after a long latent period in rabbits given a low initial dose (50–100 μc/kg.) of $^{90}$Sr, is described together with radiation dose measurements and estimations of $^{90}$Sr retention. It is suggested that accumulated radiation dose may be more important than dose rate in carcinogenesis from bone-seeking isotopes. The importance of the retention of radioactive bone-seeking isotopes in sites where there is little remodelling and only slow loss of $^{90}$Sr from the bone adjacent to sensitive tissue, is stressed. Such areas may be more important than " hot spots " away from sensitive tissues.

Proliferating squamous epithelium adjacent to bone is as much a tissue at risk from bone-seeking isotopes as the endosteal osteogenic connective tissue.

We should like to thank Leroy Scrubb for much expert technical help and Dr. Hubert Sissons for examining some of the histological material.

### REFERENCES

ALBERT, R. E., NEWMAN, W. AND ALTSHULER, B.—(1961) *Radiat. Res.*, **15**, 410.

CASARETT, G. W., TUTTLE, L. W. AND BAXTER, R. C.—(1962) *In* 'Some Aspects of Internal Irradiation' (T. F. Dougherty, ed.). Symposium, Heber, Utah 1961. New York (Pergamon Press) p. 329.

DOUGHERTY, T. F.—(1962) *Ibid.*, p. 3.
DOWNIE, E. D., MACPHERSON, S., RAMSDEN, E. N., SISSONS, H. A. AND VAUGHAN, J.—(1959) *Br. J. Cancer*, **13**, 408.
DUDLEY, R. A.—(1960) *In* 'Radiation Damage in Bone'. Vienna (International Atomic Energy Agency. p. 26.
FINKEL, M. P. AND BISKIS, B. O.—(1962) *Hlth Phys.*, **8**, 565.
FINKEL, M. P., BISKIS, B. O. AND SCRIBNER, G. M.—(1958) *Int. Conf. peaceful Uses atom. Energy*, Geneva, **22**, 65.
HASTERLIK, R. J., FINKEL, A. J. AND MILLER, C. E.—(1964) *Ann. N.Y. Acad. Sci.*, **114**, 832.
KSHIRSAGAR, S. G., LLOYD, E. AND VAUGHAN, J.—(1966) *Br. J. Radiol.*, in press.
KUZMA, J. F. AND ZANDER, G.—(1957) *Am. J. Path.*, **33**, 607.
LASKIN, S., KUSCHNER, M., ALTSHULER, B. AND NELSON, N.—(1964) *Hlth Phys.*, **10**, 1229.
LITVINOV, N. N.—(1957) *Arkh. Patol.*, **19**, Pt. 1, 26.
MACPHERSON, S., OWEN, M. AND VAUGHAN, J.—(1960) *J. Bone Jt Surg.*, **42B**, 395.—(1962) *Br. J. Radiol.*, **35**, 221.
NILSSON, A.—(1962) *Acta vet. scand.*, **3**, 1.
OWEN, M.—(1962) *In* 'Some Aspects of Internal Irradiation' (T. F. Dougherty, ed.). Symposium, Heber, Utah 1961. New York (Pergamon Press), p. 409.—(1965) *Proc. Second Eur. Symp. on Calcified Tissues*, Liege. p. 11.
OWEN, M., SISSONS, H. A. AND VAUGHAN, J.—(1957) *Br. J. Cancer*, **11**, 229.
OWEN, M. AND VAUGHAN, J.—(1959) *Br. J. Radiol.*, **32**, 714.
VAN PUTTEN, L. M. AND DE VRIES, M. J.—(1962) *J. natn. Cancer Inst.*, **28**, 587.
SISSONS, H. A. AND VAUGHAN, J.—(1960) *Nature, Lond.*, **185**, 399.
SKORYNA, S. C. AND KAHN, D. S.—(1959) *Cancer*, **12**, 306.
VAUGHAN, J.—(1962) *Int. Rev. exp. Path.*, **1**, 244.—(1965) Paper submitted to Sixth Radiobiology Forum, Medical Research Council, 'Radiation Damage to Bone'.